# Occurrence and Characteristics of *Mcr*s among Gram-Negative Bacteria Causing Bloodstream Infections of Infant Inpatients between 2006 and 2019 in China

**DOI:** 10.1128/spectrum.01938-21

**Published:** 2022-02-09

**Authors:** Lijuan Wu, Tingting Xu, Yang Ji, Jingjie Song, Feiling Wang, Junxi Huang, Kai Zhou

**Affiliations:** a Clinical Laboratory, Shenzhen Bao'an Women and Children's Hospital, Shenzhen, Guangdong, China; b Shenzhen Institute of Respiratory Diseases, Second Clinical Medical College (Shenzhen People's Hospital), Jinan University, Shenzhen, Guangdong, China; c The First Affiliated Hospital (Shenzhen People's Hospital), Southern University of Science and Technology, Shenzhen, Guangdong, China; d Maternal-Fetal Medicine Institute, Shenzhen Bao'an Women and Children's Hospital, Shenzhen, Guangdong, China; University at Albany, State University of New York

**Keywords:** bloodstream infections, infant, *mcr-1*, *mcr-9*

## Abstract

The aim of this study was to determine the occurrence of mobilized colistin resistance (*mcr*) genes in Gram-negative bacteria causing bloodstream infections of child inpatients in China. Bacteria were collected between 2006 and 2019 in a maternal and child health hospital, and *mcr* genes were screened by PCR. Five of 252 isolates were *mcr*-positive, including one *mcr-1*-positive colistin-resistant Escherichia coli isolate, two *mcr-9*-positive colistin-susceptible Salmonella enterica isolates, and two *mcr-9*-positive colistin-susceptible Enterobacter hormaechei isolates. These were obtained from two neonate and three infant patients admitted between 2009 and 2018. The E. coli isolate was obtained from a neonate aged 20 min, suggestive of a possible mother-to-neonate transmission. The five *mcr*-positive isolates were multidrug resistant, and two S. enterica and one *E. hormaechei* isolate showed a hypervirulent phenotype compared to a hypervirulent Klebsiella pneumoniae type strain in a Galleria mellonella infection model. The *mcr-1* gene was carried by an IncX4-type pA1-like epidemic plasmid, and the *mcr-9* gene was detected on IncHI2/2A-type novel plasmids co-carrying multiple resistance genes. The four IncHI2/2A-type plasmids shared a backbone and a high similarity (≥77% coverage and ≥ 90% nucleotide identity), suggesting that they were derived from a common ancestor with cross-species transmission and have circulated locally over a long period. The conjugation assay showed that the *mcr-1*-encoding plasmid and one *mcr-9*-encoding plasmid were self-transmissible to E. coli with high conjugation frequencies. Our findings demonstrate that *mcr* genes have disseminated in the community and/or hospitals, mediated by epidemic/endemic plasmids over a long period. The study shows that continuous monitoring of *mcr* genes is imperative for understanding and tackling their dissemination.

**IMPORTANCE** Antimicrobial resistance, especially the spread of carbapenemase-producing *Enterobacteriaceae* (CPE), represents one of the largest challenges to One Health coverage of environmental, animal, and human sectors. Colistin is one of the last-line antibiotics for clinical treatment of CPE. However, the emergence of the mobilized colistin resistance (*mcr*) gene largely threatens the usage of colistin in the clinical setting. In this study, we investigated the existence of *mcr* genes in 252 Gram-negative bacteria collected between 2006 and 2019 which caused bloodstream infections of child inpatients in China. We found a high prevalence of *mcr* carriage among children inpatients in the absence of professional exposure, and *mcr* might have widely disseminated in the community via different routes. This study emphasizes the importance of rational use of colistin in the One Health frame, and highlights both the urgent need for understanding the prevalence and dissemination of *mcr* genes in different populations and the importance of effective measures to control their spread.

## INTRODUCTION

With the increasing global incidence of multidrug resistant (MDR) pathogens, particularly carbapenem-resistant Gram-negative bacteria (GNB), colistin is considered one of the last-resort antibiotics for the treatment in clinical settings (1). However, the recent emergence of mobile colistin resistance (*mcr*) genes largely limits therapeutic options and compromises therapeutic efficiency in clinical settings, thus representing a threat to the health care network. Since the first report of *mcr-1* (2), this family has rapidly extended to 10 members (*mcr-1* to *mcr-10*) and has been widely identified in numerous bacterial species in six out of seven continents (3). A number of reports have detected *mcr*-positive bacteria in various clinical samples, such as feces, sputum, and blood, most of which are from adult patients. However, very few studies have addressed the occurrence of *mcr* genes in child patients. By screening fecal samples from 337 healthy school children in the Bolivian Chaco region, one study showed a high proportion of *mcr-1* carriers (38.3%) mainly belonging to Escherichia coli (171/173) (4). Another retrospective study of 12,053 Salmonella isolates, collected from outpatients with diarrhea over an 11-year duration, identified 37 *mcr-1*-positive strains, and most of the positive outpatients were aged <5 years (33/37; 89%) (5). The high prevalence of *mcr* carriage among children in the absence of professional exposure is unexpected, and emphasizes the importance of rational use of colistin. It has been suggested that the use of colistin in food animals largely contributes to the prevalence of *mcr* genes in humans and in the environment (6).

Of greater concern, a recent study reported one *mcr-1*-positive *S. Typhimurium* ST34 strain after analyzing 218 bloodstream and 110 intestinal infection samples from children (7). To our knowledge, this is the orphan study of *mcr*-1-positive strains isolated from the bloodstream in children. Bloodstream infection has been considered a major cause of neonatal morbidity and mortality in developing countries (8). Therefore, understanding the occurrence of *mcr* among bloodstream isolates has clinical significance. Here, we performed a survey on the prevalence of *mcr* genes in GNB isolates causing bloodstream infections in child patients, especially neonate patients, admitted to a maternal and child health hospital between 2006 and 2019 in China. Our results provide an insight into the epidemiological and genetic characteristics of *mcr*-positive bloodstream isolates recovered from neonate and infant patients.

## RESULTS

### Clinical data and characteristics of *mcr*-positive bloodstream isolates.

A total of 252 unduplicated bloodstream isolates were collected from 252 patients admitted to one maternal and child health hospital in Southern China between 2006 and 2019. Of these patients (96 female and 156 male), 241 (95.6%) were infants (≤1 year old), of which 213 (84.5%) were neonates (aged ≤4 weeks). The other 11 patients were 1 to 5 years old. The patients were from two different clinical wards, including 225 from the neonatology department and 27 from the pediatric department (see Table S1 in the supplemental material). Species identification assigned the bloodstream isolates as Klebsiella pneumoniae (*n* = 113), E. coli (*n* = 87), S. enterica (*n* = 25), Serratia marcescens (*n* = 21), and Enterobacter cloacae (*n* = 6). Most of the isolates were resistant to ampicillin (84.6%, 203/240), followed by piperacillin (63.7%, 149/234) and cefotaxime (49.8%, 121/243). All of the isolates were susceptible to carbapenems, and most were susceptible to amikacin (91.8%, 224/244), levofloxacin (81.1%, 198/244), and piperacillin-tazobactam (78.9%, 198/251) (Table S1).

PCR screens followed by DNA sequencing identified five *mcr*-positive isolates, including one *mcr-1*-positive E. coli isolate (Eco-569), two *mcr-9*-positive S. enterica isolates (Sal-661 and Sal-679), and two *mcr-9*-positive E. cloacae complex isolates (Ecl-683 and Ecl-686). The length of *mcr-9* gene detected in Sal-679 was approximately 2,500 bp, which was caused by an insertion of IS*1R*. Eco-569 was obtained from a neonate (aged 20 min) diagnosed with multiple diseases, including neonatal sepsis, septic shock, and peritonitis, in March 2013. Sal-661 was recovered from an 8-month-old infant with Salmonella septicemia and acute bronchitis in October 2012. Sal-679 was isolated from a 1-year-old female who was hospitalized due to fever in May 2018. Ecl-683 was isolated from a 10-day-old premature neonate in May 2009, and Ecl-686 was isolated from a 32-day-old infant in June 2014 (Table 1). All of the patients received antibiotic treatments and four of them were cured (Table 1). Antimicrobial susceptibility results showed that Eco-569 was resistant to colistin (MIC = 4 mg/L), while the *mcr-9*-harboring isolates remained susceptible to colistin (MIC = 2 mg/L) (Table 2).

**TABLE 1:** Summary of clinical information for the *mcr*-carrying isolates

Isolate	Date of isolation	Age	Gender	Diagnosis	Antibiotic usage	Outcome
Eco-569	18/03/2013	20 min	Male	Neonatal sepsis, septic shock, peritonitis, stage 4 intracranial hemorrhage, pneumorrhagia, intrauterine infectious pneumonia	Ceftazidime, meropenem, piperacillin/sulbactam, fluconazole	Transferred to another hospital after 24-day hospitalization
Sal-661	23/10/2012	8 mo and 13 days	Male	Salmonella septicemia, acute bronchitis	Ceftriaxone and ribavirin, followed by ceftriaxone and erythromycin	Cured, discharged after 5-day hospitalization
Sal-679	11/03/2018	1 yr	Female	Salmonella septicemia, acute bronchitis, Mycoplasma pneumoniae infection, myocardial damage	Ceftriaxone and azithromycin	Improved, discharged on March 19th
Ecl-683	04/05/2009	10 days	Male	Premature infant, hypoglycemia, severe anemia	Sulperazone	Cured, discharged on May 15th
Ecl-686	20/07/2014	32 days	Male	Severe pneumonia, heart failure, congenital heart disease	Cefuroxime and erythromycin used after admission; vancomycin added on July 6; ceftriaxone added on July 12, combined with acyclovir and meropenem	Cured

**TABLE 2:** MIC profiles of *mcr*-positive isolates^*a*^

Isolate	MIC (mg/L)														
	CAZ	CXM	FEP	IPM	MEM	CIP	AMP	AMK	GEN	CSL	SXT	CHL	PMB	CST	TGC
Eco-569	**16**	**>64**	8	0.25	0.0625	0.25	**>64**	4	1	**>64**	<2.5	4	2	**4**	0.125
Sal-661	**64**	**>64**	1	1	0.0625	0.125	**>64**	**>128**	**>32**	8	**>320**	**>128**	1	2	0.5
Sal-679	0.0625	4	0.0625	1	0.0625	0.0625	**>64**	8	8	4	**>320**	**>128**	1	2	0.5
Ecl-683	**32**	**>64**	1	2	0.0625	0.125	**>64**	16	**>32**	2	**>320**	**>128**	1	2	0.5
Ecl-686	8	**>64**	8	1	0.0625	0.125	**>64**	8	**>32**	16	**>320**	**>128**	1	2	0.5
EC600	0.0625	4	0.0625	0.25	0.03	0.0625	2	0.5	0.25	0.25	<2.5	2	0.5	0.5	0.125
TEC600-569	**16**	**>64**	0.0625	0.25	0.03	0.0625	**>64**	0.5	2	**>64**	<2.5	1	1	2	0.125
TEC600-679	0.0625	1	0.0625	0.25	0.03	0.0625	**>64**	0.5	2	0.25	**>320**	**64**	0.5	0.5	0.125

a CAZ, ceftazidime; CXM, cefuroxime; FEP, cefepime; IPM, imipenem; MEM, meropenem; CIP, ciprofloxacin; AMP, ampicillin; AMK, amikacin; GEN, gentamicin; CSL, cefoperazone; SXT, trimethoprim-sulfamethoxazole; CHL, chloramphenicol; PMB, polymyxin B; CST, colistin; TGC, tigecycline. Data in bold represent resistance.

### Genetic characterization of the *mcr* positive isolates.

Short-read sequencing was performed for the five *mcr*-positive isolates, and the *mcr-9* positive isolates were further sequenced by long-read sequencing (Table S2). Sal-661 and Sal-679 were serotyped as Stanley and Schwarzengrund *in silico*, respectively. Ecl-683 was classified as *E. hormaechei* subsp. *steigerwaltii*, with an average nucleotide identity (ANI) value of 98.7% compared to the type strain DSM16691 (BioSample: SAMN05581751); Ecl-686 was classified as *E. hormaechei* subsp. *xiangfangensis*, with an ANI value of 99.1% compared to the type strain LMG27195 (BioSample: SAMN05581746) (Table S3). *In silico* multilocus sequence typing (MLST) assigned Eco-569, Sal-661, Sal-679, Ecl-683, and Ecl-686 to ST4381, ST29, ST96, ST175, and ST303, respectively. Multiple antimicrobial resistance genes (ARGs) were detected in each of the isolates (Table S4). Ecl-686 and Eco-569 carried the highest (*n* = 24) and lowest (*n* = 7) numbers of ARGs, respectively. The ARGs *strA*, *strB*, *bla*_TEM-1_, *dfrA19*, *sul1*, *tet*(D), and *mcr-9* were shared by the four *mcr-9* positive isolates, and two copies of *mcr-9* were found in Ecl-686.

### Virulence of *mcr*-positive isolates.

**FIG 1:**
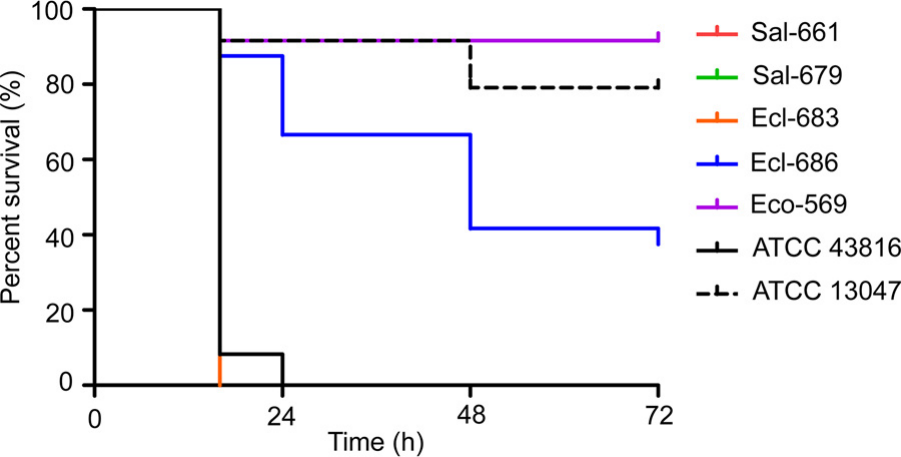
Virulence potential of *mcr*-positive strains. The virulence of five *mcr*-positive isolates was estimated by using a G. mellonella model with an inoculum of 1 × 10^6^ CFU. K. pneumoniae strain ATCC 43816 and E. cloacae subsp. *cloacae* strain ATCC 13047 were used as the hypervirulent and hypovirulent controls, respectively.

The virulence of the *mcr*-positive isolates was estimated using a Galleria mellonella larvae infection model. At 24 h postinfection at an inoculum of 1 × 10^6^ CFU, G. mellonella survival rates were 0% for Sal-661, Sal-679, and Ecl-683; 91.7% for Eco-569; and 66.7% for Ecl-686. Survival rates of 0% and 91.7% were recorded for the hypervirulent K. pneumoniae type strain ATCC 43816 and the E. cloacae strain ATCC 13047, respectively (Fig. 1). The data suggest that three of the five *mcr*-positive bloodstream isolates (Sal-661, Sal-679, and Ecl-683) are hypervirulent.

### Genetic structure of *mcr*-carrying plasmids.

**FIG 2:**
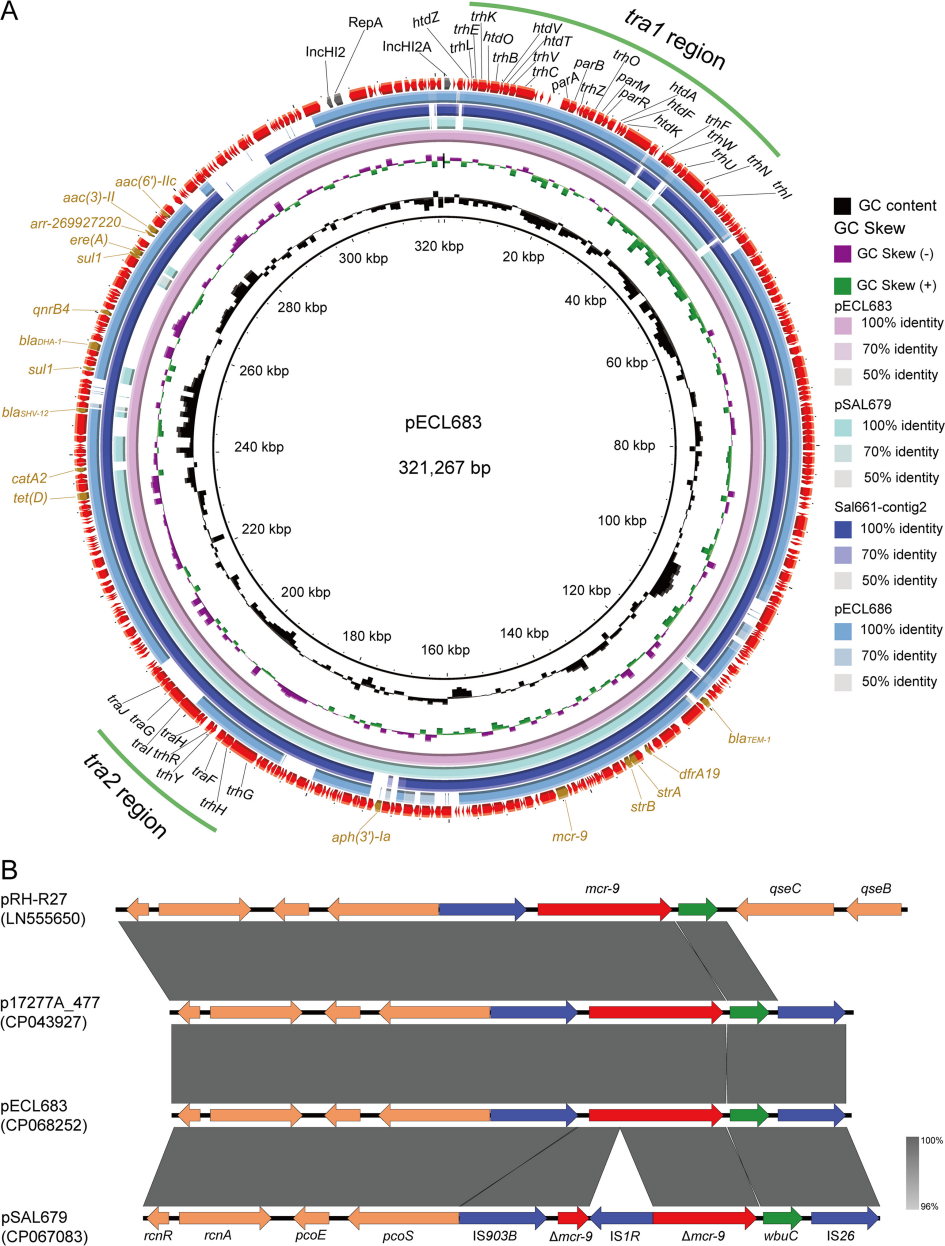
Plasmid structure and genetic context of *mcr-9*. (A) Sequence alignment of *mcr-9*-harboring plasmids pECL683, pSAL679, and pECL686, and an uncircularized contig of Sal-661. pECL683 was used as a reference. Outer circle with red arrows denotes annotation of reference plasmid. The *tra1* and *tra2* regions for conjugative transfer are indicated by green curves on the outer circle, and antibiotic resistance genes are indicated by the brown arrow. (B) Comparison of *mcr-9* regions detected in pRH-R27, p17277A_477, pECL683, and pSAL679. Gray shading denotes regions of shared homology. Arrows indicate the direction of gene transcription.

Hybrid assemblies identified that the *mcr-9* gene of Sal-679, Ecl-683, and Ecl-686 was located on a 295,452-bp (pSAL679), 321,267-bp (pECL683), and 303,535-bp (pECL686) circularized plasmid, respectively. The other copy of *mcr-9* carried by Ecl-686 was detected on the chromosome. The *mcr-9* of Sal-661 was located on an un-circularized contig encoding an IncHI2/2A-type replicon with a size of 271,919 bp; we supposed that this contig belonged to a plasmid and named it pSAL661 here (Fig. S1 in the supplemental material). All of the *mcr-9*-harboring plasmids encode an IncHI2/2A-type replicon (Table S4), suggesting that IncHI2/2A-type plasmids mediate the dissemination of *mcr-9* in the region surveyed. Except for *mcr-9*, multiple ARGs located on the *mcr-9* plasmids are mainly responsible for the MDR phenotype of the isolates (Fig. 2A). These include *bla*_DHA-1_, *bla*_SHV-12_, and *bla*_TEM-1_ for *β*-lactam resistance; *strAB*, *aac*(3)-II, and *aac*(6′)-IIc for aminoglycoside resistance; *dfrA19* for trimethoprim resistance; *sul1* for sulfonamide resistance; *tet*(D) for tetracycline resistance; *qnrB4* for quinolone resistance; and *ere*(A) for macrolide resistance. BLAST comparisons against the NCBI Nucleotide Collection (nt database) as of 6 July 2021 revealed that no homologies were found for the four *mcr-9*-encoding plasmids, suggesting that they were novel plasmids. Pairwise comparisons showed that the four plasmids were homologous to each other, with at least 76% coverage and 90% nucleotide identity (Fig. S2). They shared a similar backbone that mostly includes regions essential for plasmid replication, maintenance, and conjugative transference (Fig. 2A). These results suggest that the four plasmids were derived from a common ancestor. Given that the four isolates were obtained between 2009 and 2018, we suppose that these plasmids have mediated the cross-species dissemination of *mcr-9* gene in this region over a long period. Conjugation assays showed that only pSAL679 was self-transmissible, although multiple attempts were made for the others. The transfer frequency of pSAL679 was determined as approximately 3 × 10^−3^ per recipient cell. The colistin MIC of the pSAL679 transconjugant was 0.5 mg/L, which was identical to that of the recipient E. coli EC600 (Table 2).

**FIG 3:**
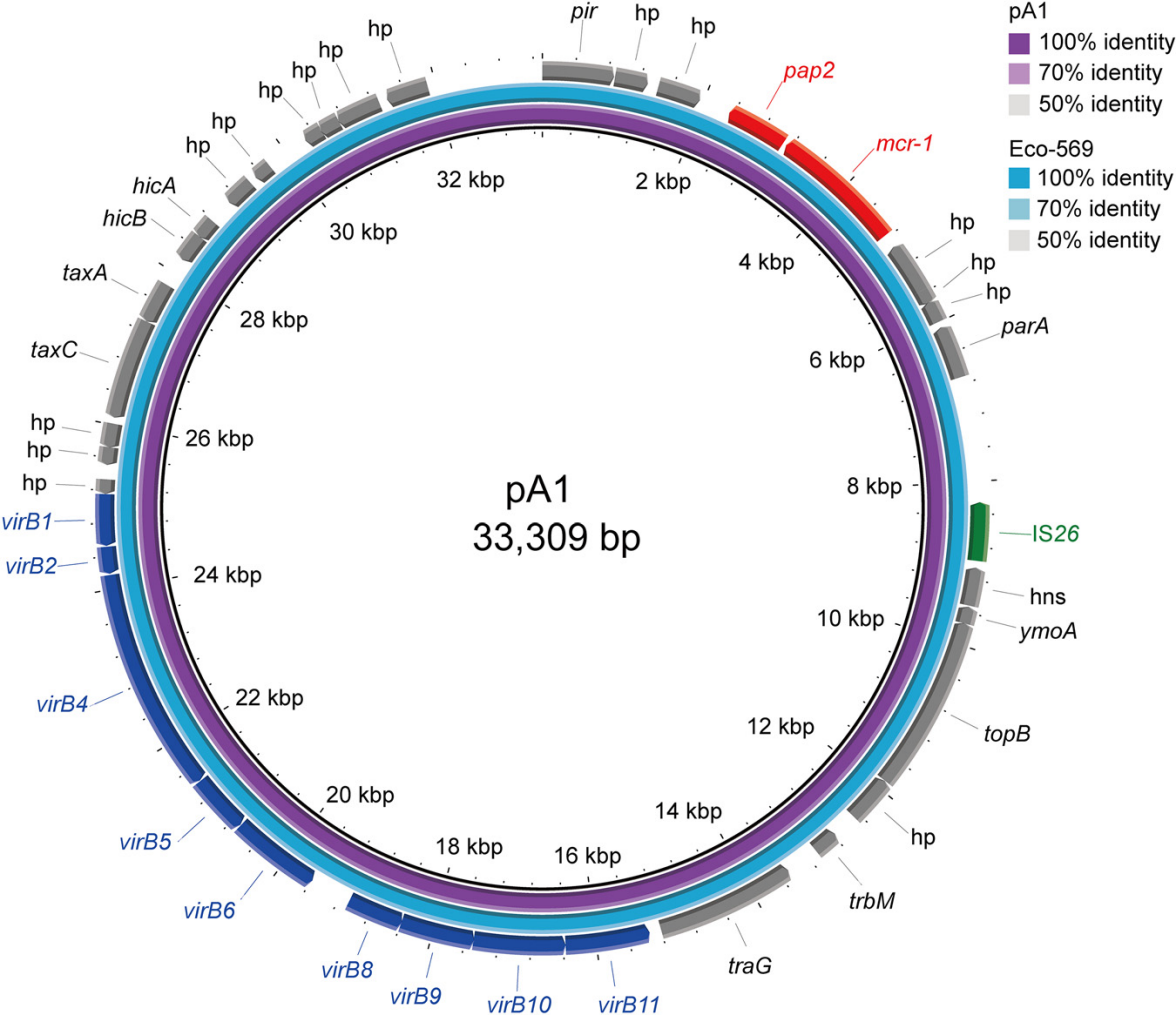
Location and genetic environment of *mcr-1*. Sequences of Eco-569 genome and *mcr-1*-harboring plasmid pA1 were aligned. pA1 was used as a reference.

The *mcr-1* gene of Eco-569 was detected on a 26,431-bp uncircularized contig. A BLAST search for the contig in GenBank showed that it was identical (100% coverage and 100% identity) to an IncX4-type plasmid pA1 (33,309 bp; accession no. LC477138) harbored by an E. coli strain A1 recovered from municipal wastewater (9). Mapping the genome sequence of Eco-569 to pA1 showed a high coverage (>99.9%) and nucleotide identity (>99.9%) (Fig. 3), indicating that the *mcr-1* plasmid carried by Eco-569 (pECO569) is pA1-like. A BLAST search for the DNA sequence of pA1 in GenBank showed that the top 100 homologous plasmids (coverage and identity of ≥99%) were all carried by *Enterobacterales*, such as E. coli, S. enterica, and *K. pneumonia*. These isolates were collected from multiple countries, indicating that the pA1-like plasmid is an epidemic plasmid in *Enterobacterales* which mediates the wide spread of *mcr-1*. The *mcr-1* plasmid pECO569 is self-transmissible into E. coli EC600, with a high transfer frequency of approximately 2 × 10^−2^ per recipient cell. The colistin MIC of the transconjugant was 4-fold higher (2 mg/L) than that of the recipient strain EC600 (Table 2).

### Genetic environment of *mcr* genes.

The genetic context of the *mcr-9* gene was identical in the four isolates, with a unique cupin fold metalloprotein-coding gene, *wbuC* downstream of *mcr-9*; additionally, the *mcr-9*-*wbuC* was surrounded upstream by an IS*903B* and downstream by an IS*26* (Fig. 2B). This genetic environment has been reported previously (10) and was found on several IncHI2 plasmids, such as p17277A_477 (accession no. CP043927). Note that the genetic environments of the two copies of *mcr-9* detected in Ecl-686 were identical, indicating that the structure “IS*903B-mcr-9*-*wbuC-*IS*26*” could be mobilizable. The genetic context of *mcr-1* was identical to that of pA1, which has been characterized by the presence of an IS*26* upstream of the *mcr-1*-*pap2* element with an 8-bp duplication (TCACACAG) (11).

## DISCUSSION

The wide dissemination of plasmid-born *mcr* genes represents a high threat to the public health network. Currently, most of the first reported *mcr* genes are associated with food animals, such as pigs, calves, and chickens (2, 12–17), indicating that the heavy use of polymyxins in veterinary medicine promotes the emergence, evolution, and dissemination of *mcr* genes. The human-impacted environment also plays a huge role in promoting the spread of *mcr* genes (18–20). Multiple studies have reported the identification of *mcr* genes in patients, even in healthy populations (21–23), suggesting that they are an important reservoir of *mcr* genes. However, the cohorts of most studies are adults, and the carriage of *mcr* genes in children, especially infants, has been much less thoroughly investigated. Additionally, most of the studies have detected *mcr*-positive isolates in fecal samples from children (24–26), and rarely in specimens with clinical significance, such as blood samples. Therefore, the conclusions derived are epidemiologically significant, but less meaningful for the guidance of clinical treatment.

In this study, we retrospectively investigated *mcr* genes among bloodstream isolates obtained from child patients during a 14-year period, most of which were neonates (84.5%). Prior to this study, an orphan study was conducted with *mcr*-positive bloodstream isolates obtained from child patients (7). Conversely, we found not only one *mcr-1*-positive isolate, but also a high proportion of *mcr-9*-positive isolates recovered from the blood cultures of two neonates and three infants. To our knowledge, this is the first report on the identification of *mcr-9* in bloodstream isolates from neonates and infants. The prevalence rates of *mcr-9* in Salmonella (8%) and E. cloacae isolates (33.3%) causing bloodstream infections reported here are much higher than those of *mcr-1* in Salmonella bloodstream isolates (0.4%) (7). The MDR profiles of these isolates further increase the difficulty of curing infections, highlighting the need for rational use of antibiotics in children. Of more concern, three of the *mcr-9*-positive isolates showed a hypervirulence phenotype compared to a typical hypervirulent K. pneumoniae strain in a G. mellonella infection model. The convergence of hypervirulence and MDR could strongly challenge current clinical treatment, especially for child patients.

Notably, all of the *mcr*-positive patients in this study were neonates and infants. We therefore suppose that these patients might have been infected through a mother/caregiver-to-infant and/or hospital-acquired route. Although we could not trace the infection routes for all patients, the *mcr-1*-positive E. coli isolate, recovered from a neonate patient aged 20 min, suggests a strong possibility of transmission from the mother. This is also a strong signal that *mcr* genes have disseminated in the community.

Following *mcr-1*, *mcr-9* is one of the most widespread *mcr* genes reported (3), and it is predominantly harbored by Enterobacter spp. and S. enterica (10), which is consistent with our findings here. Although the dissemination of *mcr-9* is mediated by diverse promiscuous plasmids across various bacterial species, current data show that IncHI2-type plasmids are its major vectors (22, 27), and they are also frequently involved in the spread of multiple ARGs (28). This is further supported by our findings that *mcr-9* is carried by IncHI2/2A plasmids co-harboring other ARGs in the four isolates, irrespective of species. Of greater concern, the four plasmids identified in this study share a high similarity (≥76% coverage and ≥ 90% nucleotide identity), indicating that they might originate from a common ancestor. Given that the four *mcr*-*9*-positive isolates were detected over a long period (between 2009 and 2018), we suppose that these *mcr-9*-encoding IncHI2/2A plasmids might have circulated locally in the communities and/or hospitals. Controlling the dissemination of these plasmids could be important for tackling the *mcr-9* spread at the regional level. In our study, the isolates’ susceptibility to colistin (MIC = 2 mg/L) was probably due to the absence of the two-component system *qseB*/*qseC*, located downstream of *mcr-9*, which has been shown to be critical for the inducibility of *mcr-9* (29). Although *mcr-9* mediates low-level colistin resistance, we cannot exclude the possibility that the resistance of this gene could be enhanced with continuous evolution. Sustained surveillance is needed to monitor the *mcr* genes in case of their transmission.

### Limitations of the study.

One limitation of this study is that the weak activity of *mcr-9* against colistin hinders the phenotypic detection of additional *mcr-9*-harboring bacteria. Additionally, PCR detection is not a cost- or time-efficient method, which highly challenges routine surveillance. In addition, we cannot track the existence of *mcr* genes from surroundings or from mother/caregiver of positive patients due to the limitation of metadata and samples.

## MATERIALS AND METHODS

### Clinical isolates collection, *mcr* gene detection, and antimicrobial susceptibility testing.

A total of 252 unduplicated GNB strains were recovered from blood samples of child patients (aged <5 years old) admitted to a maternal and child health hospital between 2006 and 2019 in China. All of the isolates were identified using Vitek MS (bioMérieux, Marcy-l’Étoile, France). Salmonella serotyping was conducted according to the White-Kauffmann-Le Minor scheme (9th edition).

The isolates were subjected to PCR to screen *mcr* genes (*mcr-1* ∼ *mcr-10*) using primers as previously described (18). PCR-positive products were sequenced to determine the genes present. Antimicrobial susceptibility was evaluated for the *mcr*-positive isolates following CLSI guidelines (M100-S30), and the results were interpreted according to CLSI instructions, except that colistin and tigecycline resistance was defined according to EUCAST (version 10.0) clinical breakpoints.

### Whole-genome sequencing and bioinformatics analysis.

Genomic DNA was extracted from *mcr-*positive isolates using a Gentra Puregene Yeat/Bact. Kit (Qiagen, CA, United States). Whole-genome sequencing (WGS) was performed on an Illumina NovaSeq 6000 System (Illumina, San Diego, United States) generating 150-bp paired-end reads. Raw reads were trimmed using Trimmomatic (30) followed assembly with the use of SPAdes v3.12.0 (31). Long-read sequencing was performed on a Nanopore PromethION platform (Nanopore, Oxford, UK) following a 10-kbp library protocol. Long and short sequencing reads were assembled by hybrid assembly using Unicycler v0.4.8 (32), and were annotated using the RAST server (https://rast.nmpdr.org/rast.cgi). Plasmid circularity was confirmed by PCR and Sanger sequencing. The primers used in this study are listed in Table S5.

ARGs were identified by using ABRicate v0.9.8 to query the ResFinder v3.2 database (33). Plasmid replicon typing was performed using PlasmidFinder v2.1 (34). Species identifications were confirmed by calculating ANI values with a cutoff of 95% (35). *In silico* Salmonella serotyping was performed using the assembled genomic contigs and the SeqSero2 v1.1.1 tool (36). *In silico* multilocus sequence typing (MLST) was performed using PubMLST (https://pubmlst.org/). Linear comparisons of genetic context were performed using Easyfig v2.2.2 (37). Alignments were performed using BLAST Ring Image Generator (BRIG) (38, 39).

### Conjugation assay.

Conjugation was carried out using the mixed broth method, as performed in a previous study, with minor modifications (40). In brief, *mcr*-harboring isolates were used as the donors, and E. coli strain EC600 served as a recipient. The transconjugants were selected on LB agar plates containing 2 mg/L colistin and 600 mg/L rifampicin. Positive transconjugants were validated using Vitek MS to confirm the species and PCR to detect *mcr* genes. Due to thermal susceptibility, we performed the IncHI2-type plasmid conjugation assay at 30°C (41). Conjugation frequencies were expressed as the number of transconjugants per recipient cell (42).

### Galleria mellonella infection assay.

The virulence of *mcr*-positive isolates was estimated using a G. mellonella model. Larvae weighing about 300 mg were selected for the assay. Overnight cultures were washed and further adjusted with phosphate-buffered saline. Eight larvae in each group were infected with bacteria in a 1 × 10^6^ CFU inoculum and incubated at 37°C. The survival rate of the larvae was recorded over 72 h. A K. pneumoniae strain ATCC 43816 (43) and E. cloacae subsp. *cloacae* strain ATCC 13047 (44) were used as the hyper-virulence and low-virulence controls, respectively. The assay was performed in triplicate. Survival curves were generated using Prism6 (GraphPad Software).

### Accession number.

WGS data for Eco-569, Sal-661, Sal-679, Ecl-683, and Ecl-686 have been deposited in GenBank under accession no. JAHBAI000000000, CP067078-CP067081, CP067082-CP067085, CP068251-CP068252, and JAESUX000000000, respectively.
