# Supplementary material for: Occurrence and Characteristics of Mcrs among Gram-Negative Bacteria Causing Bloodstream Infections of Infant Inpatients between 2006 and 2019 in China
Source: Microbiol Spectr. 2022 Feb 9;10(1):e01938-21. doi: 10.1128/spectrum.01938-21 (PMC8826862; doi:10.1128/spectrum.01938-21)
Supplement: SUPPLEMENTAL FILE 2 — Supplemental material. Download SPECTRUM01938-21_Supp_1_seq6.pdf, PDF file, 0.7 MB [file spectrum01938-21_supp_1_seq6.pdf]

Supplemental figure legends

Figure S1 Sequence alignment of *mcr-9*-harboring plasmids pECL683, pSAL679, pECL686, and whole genome sequence of Sal-661. pSAL679, pECL683, and pECL686 were used as reference, respectively.

Figure S2 Comparison of three circularized *mcr-9*-carrying plasmids and one uncircularized *mcr-9*-carrying contig. The heat map shows the percentage of bases in each plasmid/contig that could be aligned to each of the other plasmids/contig. The cutoff of identity was 90%.

Table S2. Assembly statistics of the genomes of *mcr*-positive isolates.

Isolate	G+C content (%)	Length (bp)	Contigs	Longest contig (bp)	N50 (bp)	Number of contigs longer than 10 Kbp
Eco-569	50.54%	5,573,276	117	290,422	149,177	57
Ecl-683	54.87%	5,394,350	2	5,073,083	321,267	2
Ecl-686	54.76%	5,002,936	8	3,501,614	1,450,846	3
Sal-661	51.91%	5,017,993	4	4,714,141	271,919	3
Sal-661	51.85%	4,963,044	4	4,664,088	295,452	2

Table S4. Plasmid replicons and antimicrobial resistance genes detected in the *mcr*-positive isolates.

Isolate	Contig number	Start	End	Inc type	Coverage (%)	Identity (%)	Accession number	Feature detection tool	Database	Local alignment algorithm[a]
**Plasmid replicons**										
Eco-569	29	66062	66203	IncI1_1	100	100	AP005147	ABRicate	PlasmidFinder (Nucleotide)	blastn
	31	46112	46373	IncFII	100	95.8	AJ851089	ABRicate	PlasmidFinder (Nucleotide)	blastn
	34	4594	4915	IncI2	100	97.83	AP002527	ABRicate	PlasmidFinder (Nucleotide)	blastn
	47	4488	4641	Col156_1	100	94.16	NC_009781	ABRicate	PlasmidFinder (Nucleotide)	blastn
	47	9653	9806	Col156_1	100	98.05	NC_009781	ABRicate	PlasmidFinder (Nucleotide)	blastn
	57	6443	7124	IncFIB	100	97.21	AP001918	ABRicate	PlasmidFinder (Nucleotide)	blastn
	61	5472	5845	IncX4_1	100	100	CP002895	ABRicate	PlasmidFinder (Nucleotide)	blastn
	64	101	220	ColRNAI_1	90.77	89.17	DQ298019	ABRicate	PlasmidFinder (Nucleotide)	blastn
	80	2152	2413	Col(MG828)_1	100	91.6	NC_008486	ABRicate	PlasmidFinder (Nucleotide)	blastn
	83	36	295	Col(MG828)	99.24	96.92	NC_00848	ABRicate	PlasmidFinder	blastn

				_1			6		(Nucleotide)	
Sal-661	2	244153	244782	IncHI2A	100	100	BX664015	ABRicate	PlasmidFinder (Nucleotide)	blastn
	2	258436	259030	RepA_1_pKPC-CAV1321	99.66	80.37	CP011611	ABRicate	PlasmidFinder (Nucleotide)	blastn
	2	259648	259974	IncHI2	100	100	BX664015	ABRicate	PlasmidFinder (Nucleotide)	blastn
Sal-679	2	211	840	IncHI2A	100	100	BX664015	ABRicate	PlasmidFinder (Nucleotide)	blastn
	2	280471	280797	IncHI2	100	100	BX664015	ABRicate	PlasmidFinder (Nucleotide)	blastn
	2	281415	282009	RepA_1_pKPC-CAV1321	99.66	80.37	CP011611	ABRicate	PlasmidFinder (Nucleotide)	blastn
	3	258	368	Col440I	97.37	92.79	CP023920.1	ABRicate	PlasmidFinder (Nucleotide)	blastn
Ecl-683	2	211	840	IncHI2A	100	99.84	BX664015	ABRicate	PlasmidFinder (Nucleotide)	blastn
	2	305061	305387	IncHI2	100	100	BX664015	ABRicate	PlasmidFinder (Nucleotide)	blastn
	2	306005	306599	RepA_1_pKPC-CAV1321	99.66	80.37	CP011611	ABRicate	PlasmidFinder (Nucleotide)	blastn
Ecl-686	1	191220	191745	IncHI2A	83.49	100	BX664015	ABRicate	PlasmidFinder (Nucleotide)	blastn
	1	206730	207324	RepA_1_pKPC-CAV1321	99.66	80.37	CP011611	ABRicate	PlasmidFinder (Nucleotide)	blastn
	1	207942	208268	IncHI2	100	100	BX664015	ABRicate	PlasmidFinder	blastn

									(Nucleotide)	
**Antimicrobial resistance genes**										
Eco-569	29	71660	72535	*bla*$_{\text{CTX-M-55}}$	100	100	NG_049006.1	ABRicate	ResFinder (Nucleotide)	blastn
	32	17413	18546	*bla*$_{\text{EC}}$	100	98.33	NG_047496.1	ABRicate	ResFinder (Nucleotide)	blastn
	45	1730	3355	*mcr-1.1*	100	100	NG_050417.1	ABRicate	ResFinder (Nucleotide)	blastn
	5	120782	121981	*tet(A)*	100	100	NG_048154.1	ABRicate	ResFinder (Nucleotide)	blastn
	5	123059	123895	*strB*	100	100	NG_047464.1	ABRicate	ResFinder (Nucleotide)	blastn
	5	123895	124722	*strA*	100	100	NG_056002.2	ABRicate	ResFinder (Nucleotide)	blastn
	5	124759	125574	*sul2*	100	100	NG_051852.1	ABRicate	ResFinder (Nucleotide)	blastn
Sal-661	2	77597	78457	*bla*$_{\text{TEM-1}}$	100	100	NG_050145.1	ABRicate	ResFinder (Nucleotide)	blastn
	2	88402	88971	*dfrA19*	100	100	NG_050403.1	ABRicate	ResFinder (Nucleotide)	blastn
	2	90721	91548	*strA*	100	99.88	NG_056002.2	ABRicate	ResFinder (Nucleotide)	blastn
	2	91548	92384	*strB*	100	100	NG_047464.1	ABRicate	ResFinder (Nucleotide)	blastn
	2	101225	101866	*catA2*	100	100	NG_047596.1	ABRicate	ResFinder (Nucleotide)	blastn

	2	103478	104662	*tet*(D)	100	99.92	NG_048184.1	ABRicate	ResFinder (Nucleotide)	blastn
	2	187914	189533	*mcr-9.1*	100	100	NG_064792.1	ABRicate	ResFinder (Nucleotide)	blastn
	2	199133	199687	*aac(6')-Ib-AKT*	100	99.82	NG_052358.1	ABRicate	ResFinder (Nucleotide)	blastn
	2	203151	204011	*bla*$_{\text{SHV-12}}$	100	100	NG_050590.1	ABRicate	ResFinder (Nucleotide)	blastn
	3	4585	5724	*bla*$_{\text{DHA-1}}$	100	100	NG_049055.1	ABRicate	ResFinder (Nucleotide)	blastn
	3	7301	8140	*sul1*	100	100	NG_048082.1	ABRicate	ResFinder (Nucleotide)	blastn
	3	11517	12098	*aac(6')-IIc*	100	100	NG_047273.1	ABRicate	ResFinder (Nucleotide)	blastn
	3	14183	14992	*aac(3)-II*	100	100	NG_047231.1	ABRicate	ResFinder (Nucleotide)	blastn
	3	15120	15533	*arr-269927220*	100	100	NG_047480.1	ABRicate	ResFinder (Nucleotide)	blastn
	3	16395	17454	*ere*(A)	86.31	99.44	NG_047763.1	ABRicate	ResFinder (Nucleotide)	blastn
	3	17978	18817	*sul1*	100	100	NG_048082.1	ABRicate	ResFinder (Nucleotide)	blastn
Sal-679	2	113157	114017	*bla*$_{\text{TEM-1}}$	100	100	NG_050145.1	ABRicate	ResFinder (Nucleotide)	blastn
	2	123962	124531	*dfrA19*	100	100	NG_050403.1	ABRicate	ResFinder (Nucleotide)	blastn

	2	127347	128174	*strA*	100	99.88	NG_056002.2	ABRicate	ResFinder (Nucleotide)	blastn
	2	128174	129010	*strB*	100	100	NG_047464.1	ABRicate	ResFinder (Nucleotide)	blastn
	2	137840	140236	*mcr-9.1*	100	100	NG_064792.1	Blast	Nt[b]	blastn
	2	166332	167147	*aph(3')-Ia*	100	100	NG_047440.1	ABRicate	ResFinder (Nucleotide)	blastn
	2	230407	231591	*tet*(D)	100	99.92	NG_048184.1	ABRicate	ResFinder (Nucleotide)	blastn
	2	237485	238699	*floR*	100	100	NG_047877.1	ABRicate	ResFinder (Nucleotide)	blastn
	2	239655	240491	*strB*	100	100	NG_047464.1	ABRicate	ResFinder (Nucleotide)	blastn
	2	240491	241318	*strA*	100	100	NG_056002.2	ABRicate	ResFinder (Nucleotide)	blastn
	2	241355	242170	*sul2*	100	100	NG_051852.1	ABRicate	ResFinder (Nucleotide)	blastn
	2	246534	247373	*sul1*	100	100	NG_048082.1	ABRicate	ResFinder (Nucleotide)	blastn
	2	247878	248669	*aadA2*	100	100	NG_051846.1	ABRicate	ResFinder (Nucleotide)	blastn
	2	251954	253144	*tet*(C)	100	100	NG_048177.1	ABRicate	ResFinder (Nucleotide)	blastn
Ecl-683	1	492904	494049	*bla*$_{ACT-74}$	100	99.83	NG_066690.1	ABRicate	ResFinder (Nucleotide)	blastn

	1	707457	707882	*fosA*	100	95.31	NG_050405.1	ABRicate	ResFinder (Nucleotide)	blastn
	1	2464818	2465633	*aph(3')-Ia*	100	100	NG_047440.1	ABRicate	ResFinder (Nucleotide)	blastn
	2	119303	120163	*bla*$_{TEM-1}$	100	100	NG_050145.1	ABRicate	ResFinder (Nucleotide)	blastn
	2	130108	130677	*dfrA19*	100	100	NG_050403.1	ABRicate	ResFinder (Nucleotide)	blastn
	2	132427	133254	*strA*	100	99.88	NG_056002.2	ABRicate	ResFinder (Nucleotide)	blastn
	2	133254	134090	*strB*	100	100	NG_047464.1	ABRicate	ResFinder (Nucleotide)	blastn
	2	142920	144539	*mcr-9.1*	100	100	NG_064792.1	ABRicate	ResFinder (Nucleotide)	blastn
	2	170635	171450	*aph(3')-Ia*	100	100	NG_047440.1	ABRicate	ResFinder (Nucleotide)	blastn
	2	234710	235894	*tet*(D)	100	99.92	NG_048184.1	ABRicate	ResFinder (Nucleotide)	blastn
	2	237506	238147	*catA2*	100	100	NG_047596.1	ABRicate	ResFinder (Nucleotide)	blastn
	2	246662	247522	*bla*$_{SHV-12}$	100	100	NG_050590.1	ABRicate	ResFinder (Nucleotide)	blastn
	2	252510	253349	*sul1*	100	100	NG_048082.1	ABRicate	ResFinder (Nucleotide)	blastn
	2	254926	256065	*bla*$_{DHA-1}$	100	100	NG_049055.1	ABRicate	ResFinder (Nucleotide)	blastn

	2	260189	260833	*qnrB4*	100	100	NG_050502.1	ABRicate	ResFinder (Nucleotide)	blastn
	2	269551	270390	*sul1*	100	100	NG_048082.1	ABRicate	ResFinder (Nucleotide)	blastn
	2	270914	271973	*ere*(A)	86.31	99.44	NG_047763.1	ABRicate	ResFinder (Nucleotide)	blastn
	2	272835	273248	*arr-269927220*	100	100	NG_047480.1	ABRicate	ResFinder (Nucleotide)	blastn
	2	273376	274185	*aac(3)-II*	100	100	NG_047231.1	ABRicate	ResFinder (Nucleotide)	blastn
	2	276270	276851	*aac(6')-IIc*	100	100	NG_047273.1	ABRicate	ResFinder (Nucleotide)	blastn
Ecl-686	1	50159	51046	*bla*$_{\text{SFO-1}}$	100	100	NG_049982.1	ABRicate	ResFinder (Nucleotide)	blastn
	1	65634	66555	*mph(A)*	100	99.67	NG_047986.1	ABRicate	ResFinder (Nucleotide)	blastn
	1	219987	220826	*sul1*	100	100	NG_048082.1	ABRicate	ResFinder (Nucleotide)	blastn
	1	222403	223542	*bla*$_{\text{DHA-1}}$	100	100	NG_049055.1	ABRicate	ResFinder (Nucleotide)	blastn
	1	227664	228308	*qnrB4*	100	100	NG_050502.1	ABRicate	ResFinder (Nucleotide)	blastn
	1	237025	237864	*sul1*	100	100	NG_048082.1	ABRicate	ResFinder (Nucleotide)	blastn
	1	238388	239447	*ere(A)*	86.31	99.44	NG_047763.1	ABRicate	ResFinder (Nucleotide)	blastn

	1	240309	240722	*arr-269927220*	100	100	NG_047480.1	ABRicate	ResFinder (Nucleotide)	blastn
	1	240851	241660	*aac(3)-II*	100	100	NG_047231.1	ABRicate	ResFinder (Nucleotide)	blastn
	1	243745	244326	*aac(6')-IIc*	100	100	NG_047273.1	ABRicate	ResFinder (Nucleotide)	blastn
	1	247730	248371	*catA2*	100	100	NG_047596.1	ABRicate	ResFinder (Nucleotide)	blastn
	1	249982	251166	*tet(D)*	100	99.92	NG_048184.1	ABRicate	ResFinder (Nucleotide)	blastn
	1	253797	254657	*aac(3)-IId*	100	100	NG_047251.1	ABRicate	ResFinder (Nucleotide)	blastn
	1	259628	260488	*bla*$_{TEM-1}$	100	100	NG_050145.1	ABRicate	ResFinder (Nucleotide)	blastn
	1	269487	270056	*dfrA19*	100	100	NG_050403.1	ABRicate	ResFinder (Nucleotide)	blastn
	1	271806	272633	*strA*	100	99.88	NG_056002.2	ABRicate	ResFinder (Nucleotide)	blastn
	1	272633	273469	*strB*	100	100	NG_047464.1	ABRicate	ResFinder (Nucleotide)	blastn
	1	282297	283916	*mcr-9.1*	100	100	NG_064792.1	ABRicate	ResFinder (Nucleotide)	blastn
	2	1725904	1727050	*bla*$_{ACT-45}$	100	99.83	NG_050708.1	ABRicate	ResFinder (Nucleotide)	blastn
	2	1894212	1894637	*fosA*	100	95.78	NG_050405.1	ABRicate	ResFinder (Nucleotide)	blastn

	2	3535026	3535613	*catA1*	88.33	68.98	NG_051704.1	ABRicate	ResFinder (Nucleotide)	blastn
	2	3574529	3576149	*mcr-9.1*	100	99.94	NG_064792.1	ABRicate	ResFinder (Nucleotide)	blastn
	2	4615091	4618210	*oqxB9*	98.95	89.42	NG_050458.1	ABRicate	ResFinder (Nucleotide)	blastn
	2	4618234	4619409	*oqxA9*	100	86.73	NG_050427.1	ABRicate	ResFinder (Nucleotide)	blastn

[a]Nucleotide blast with minimum identity thresholds of 80 and 75% and minimum coverage thresholds of 60 and 50%, for PlasmidFinder and ResFinder, respectively.

[b]*mcr-9* was inserted by an IS*1*.

Table S5. Primers used in this study.

Primer name	Sequence (5'-3')	Product size (bp)
SAL661-contig2-F	GCCATCATCCGAACCAGTGT	570
SAL661-contig2-R	TTGAACCGCAGCCGAGAAG	
SAL679-contig2-F	ACCCACTTTCATTTCCGTTTGC	437
SAL679-contig2-R	GAACCGACTTGGACGAGTTAGG	
ECL683-contig2-F	ACCCACTTTCATTTCCGTTTGC	530
ECL683-contig2-R	GCTCATCAGAGGCATCAACTTC	
ECL686-contig1-F	AGTTACGGATGGTTTCAACG	942
ECL686-contig1-R	AGGCGTTAGCCAATCAAAGT	

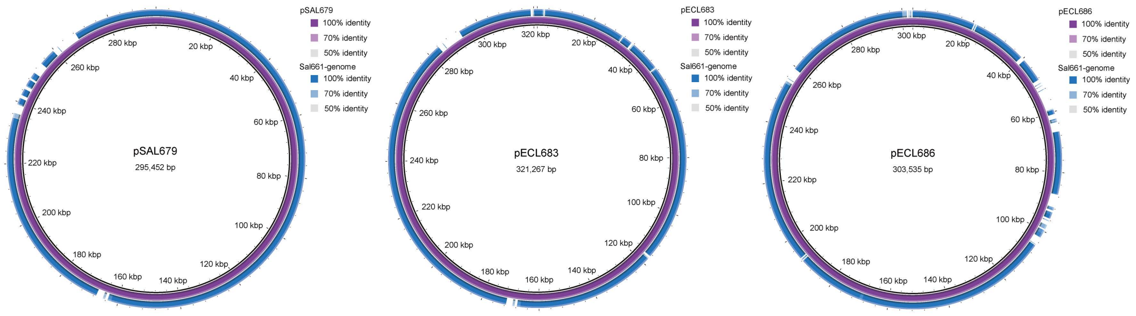

pSAL679
295,452 bp
20 kbp
40 kbp
60 kbp
80 kbp
100 kbp
120 kbp
140 kbp
160 kbp
180 kbp
200 kbp
220 kbp
240 kbp
260 kbp
280 kbp
pSAL679
100% identity
70% identity
50% identity
Sal661-genome
100% identity
70% identity
50% identity
pECL683
321,267 bp
20 kbp
40 kbp
60 kbp
80 kbp
100 kbp
120 kbp
140 kbp
160 kbp
180 kbp
200 kbp
220 kbp
240 kbp
260 kbp
280 kbp
300 kbp
320 kbp
pECL683
100% identity
70% identity
50% identity
Sal661-genome
100% identity
70% identity
50% identity
pECL686
303,535 bp
20 kbp
40 kbp
60 kbp
80 kbp
100 kbp
120 kbp
140 kbp
160 kbp
180 kbp
200 kbp
220 kbp
240 kbp
260 kbp
280 kbp
300 kbp
pECL686
100% identity
70% identity
50% identity
Sal661-genome
100% identity
70% identity
50% identity

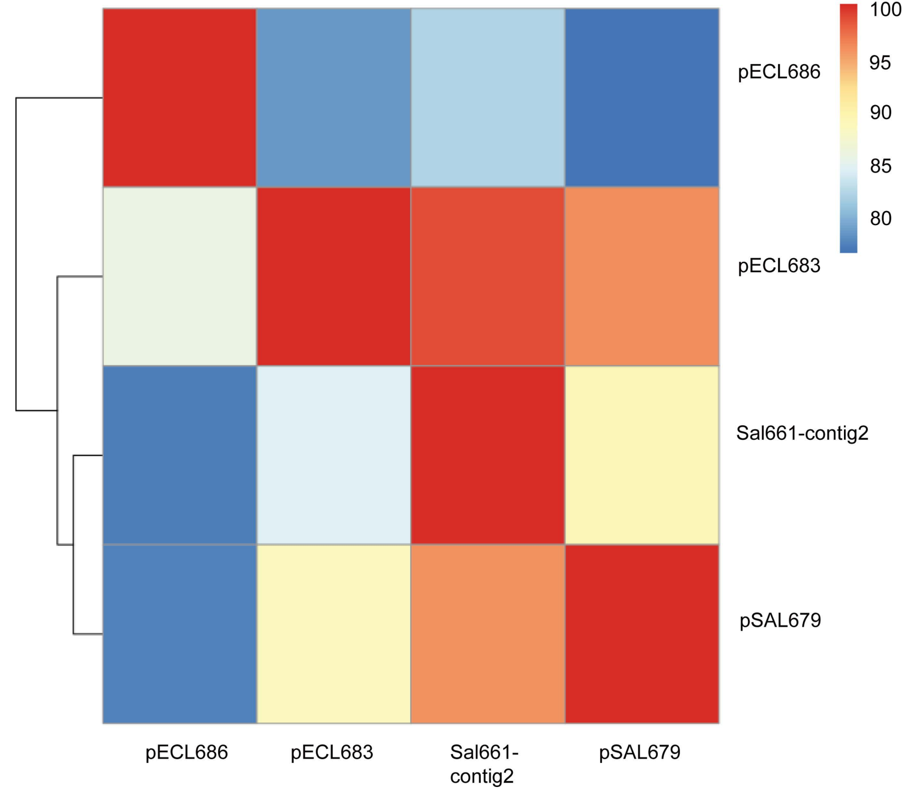
100
95
90
85
80
pECL686
pECL683
Sal661-contig2
pSAL679
pECL686
pECL683
Sal661-
contig2
pSAL679
